# Scientists’ deficit perception of the public impedes their behavioral intentions to correct misinformation

**DOI:** 10.1371/journal.pone.0287870

**Published:** 2023-08-02

**Authors:** Sera Choi, Ashley A. Anderson, Shelby Cagle, Marilee Long, Nicole Kelp

**Affiliations:** 1 School of Communications, Grand Valley State University, Allendale, Michigan, United States of America; 2 Department of Journalism and Media Communication, Colorado State University, Fort Collins, Colorado, United States of America; 3 Department of Microbiology, Immunology, and Pathology, Colorado State University, Fort Collins, Colorado, United States of America; National Sun Yat-sen University, TAIWAN

## Abstract

This paper investigates the relationship between scientists’ communication experience and attitudes towards misinformation and their intention to correct misinformation. Specifically, the study focuses on two correction strategies: source-based correction and relational approaches. Source-based approaches combatting misinformation prioritize sharing accurate information from trustworthy sources to encourage audiences to trust reliable information over false information. On the other hand, relational approaches give priority to developing relationships or promoting dialogue as a means of addressing misinformation. In this study, we surveyed 416 scientists from U.S. land-grant universities using a self-report questionnaire. We find that scientists’ engagement in science communication activities is positively related to their intention to correct misinformation using both strategies. Moreover, the scientists’ attitude towards misinformation mediates the relationship between engagement in communication activities and intention to correct misinformation. The study also finds that the deficit model perception–that is, the assumption that scientists only need to transmit scientific knowledge to an ignorant public in order to increase understanding and support for science–moderates the indirect effect of engagement in science communication activities on behavioral intention to correct misinformation using relational strategies through attitude towards misinformation. Thus, the deficit model perception is a barrier to engaging in relational strategies to correct misinformation. We suggest that addressing the deficit model perception and providing science communication training that promotes inclusive worldviews and relational approaches would increase scientists’ behavioral intentions to address misinformation. The study concludes that scientists should recognize their dual positionality as scientists and members of their community and engage in respectful conversations with community members about science.

## Introduction

In recent years, concerns over misinformation have become widespread, especially in light of the high cost of the COVID-19 pandemic and vaccine hesitance in terms of both economic and morbidity burden [1,2]. The responsibility for preventing and stopping the spread of misinformation has been placed on a variety of stakeholders, including media producers, educators, health professionals, researchers, and funders [3]. These calls to action are more likely to succeed if we understand who is susceptible to misinformation, which techniques are effective in responding to misinformation, and how to motivate individuals to adopt them.

Progress has been made on two of these three fronts. We know more about who is susceptible to misinformation and why [e.g., 4,5] and which techniques are effective for correcting misinformation [6,7]. Research that identifies who addresses misinformation, why they do so, and what corrective techniques they use in different circumstances is limited but growing [8]. As calls for scientists to correct misinformation grow [9,10], it becomes increasingly important to understand the factors that increase scientists’ likelihood of engaging in these misinformation correction activities.

Scientists’ engagement with the public plays a critical role in addressing societal challenges [11]. Engagement activities include working at open-house events or science festivals, participating in public meetings, meeting with policymakers, delivering lectures to nonexpert audiences, writing blogs, giving interviews to journalists and engaging on social media (e.g., writing about topics related to their research, participating in discussions, etc.) [12]. Existing scholarship has examined the factors that influence scientists’ public engagement activities [13–15]; however, little research has specifically investigated the relationship between scientists’ participation in engagement activities and their willingness to correct misinformation. Through their experience with science communication activities, scientists may be more willing to participate in societal communication challenges such as correcting misinformation.

Individuals’ behavioral intentions to correct misinformation may be influenced by different factors. Previous studies have empirically examined the relationship between attitudes and behavioral intentions related to addressing misinformation in various contexts such as correcting COVID-19 rumors [16], sharing unverified information [17], and verifying the information before disseminating it [18]. Here, we examine the mediating role of attitude toward misinformation (i.e., concerns over misinformation) in the relationship between scientists’ communication activities and their misinformation correction behavioral intentions.

Many approaches for correcting misinformation involve developing audience members’ skills related to identifying misinformation or providing corrective information from trusted sources. The goal of such strategies is to combat misinformation by helping people hone their analytical skills, such as logically examining the material in question and critically evaluating the information source [19]. More recently, researchers have investigated relational approaches that prioritize fostering relationships or dialogue as a response to key science communication challenges [20]. These relational approaches provide another important tool to address misinformation. Thus, this study aims to provide a more granular understanding of the drivers of scientists’ willingness to partake in correcting misinformation using both source-based and relational strategies.

The deficit model perception, or the attitude that the public does not know enough about science, likely plays a role in scientists’ willingness to correct misinformation. While studies have shown that scientists continue to hold deficit model perceptions when communicating with non-scientist audiences [21,22], little is known about how the deficit model perception predicts their likelihood to engage in misinformation correction. Using a survey of scientists at land-grant universities in the United States, we examine the dynamics of past communication behavior along with two key attitudes–attitude toward misinformation (i.e., concern over misinformation) and the deficit model perception–to predict scientists’ likelihood of engaging in misinformation correction using both source-based and relational approaches.

## The state of misinformation

Misinformation has been defined by many scholars, and these definitions often emphasize the audience member’s role. For instance, Kuklinski and colleagues state that misinformation refers to circumstances when “people hold inaccurate beliefs, and do so confidently” [23 pp792]. Likewise, Lewandowsky et al. define misinformation as “any piece of information that is initially processed as valid but is subsequently retracted or corrected” [24 pp124-125]. Building on these two definitions, for this study, we define misinformation as information that people perceive to be accurate when it is not. We also conceptualize misinformation as distinct from disinformation, or the purposeful act of sharing misleading information [25].

Recent research highlights a growing emphasis on identifying effective ways to correct misinformation, often through “the presentation of information designed to rebut an inaccurate claim or a misperception” [26]. An individual may be exposed to misinformation correction by witnessing correction, correcting others, or being corrected [8]. Examples of corrective behaviors include citing a credible source [27,28], debunking, and prebunking [29,30]. Most of these strategies have been tested almost exclusively in social media spaces, where significant amounts of exposure to and correction of misinformation occurs [26,31].

### Strategies to correct misinformation

#### Source-based strategies

Source-based approaches to addressing misinformation emphasize sharing corrective information from trusted sources in a manner that encourages audiences to believe the trusted, accurate information over misinformation. Such approaches are grounded in the principles of cognitive processing. Who people are (e.g., audience characteristics), the views they hold about a topic (e.g., attitudes or levels of knowledge), and the cues they rely upon when selecting an information source (e.g., likeability) contribute to the effectiveness of misinformation corrective techniques [19,32]. People’s susceptibility to misinformation can be reduced before they encounter the misinformation. Techniques focus on involve developing media literacy skills to check the source or prompting reflection on red flags in the message (a cognitive audience characteristic) [27]. Prebunking involves a type of knowledge change among audience members. Prebunking shows people examples of misinformation so that they will be better equipped to spot it and question it when they encounter it [29,30]. Other source-based approaches are focused on correcting misinformation that has already surfaced; these are often based on audience perceptions of the corrective source. Testing in the social media environment shows that when someone sees another person calling out misinformation while posting a link to a trusted source, misperceptions do not prevail [33].

Observational corrective techniques assume that institutional sources of information (e.g., news media, government) are best for mitigating misperceptions [33]. Yet, the ability of institutionally based sources to reach publics is limited. For example, trust in the CDC, one of the widely tested sources of information in source-based misinformation correction studies, has decreased since the start of the Covid-19 pandemic [34]. A growing lack of trust in such institutional sources indicates addressing misinformation will require community approaches grounded in relationship-building and interpersonal trust [35].

#### Relational strategies

Science communication practitioners are increasingly calling upon scientists and organizations to use community-based approaches involving a variety of stakeholders to address science communication challenges [36]. Public health community leaders, such as those who work in county public health departments, often engage in dialogic approaches to misinformation correction that emphasize relationship-building and listening to individual concerns during person-to-person conversations [20]. Relational approaches promote a shared sense of community, which may drive the efficacy of responses to misinformation and the prevention of misinformation [37].

Relational approaches may work well when the misinformation aligns with people’s values [26]. People tend to accept information that supports or aligns with their preexisting beliefs, values, or notions, so they are more likely to believe misinformation when it aligns with those values [38]. Relational approaches may be most effective in overcoming misinformation bound within the dynamics of motivated reasoning because relational approaches are based on an interaction that allows for listening, acknowledgement of others’ perspectives and values, and tolerance of difference. Dialogic activities build trust [39]. And people accept information that comes from a perceived trusted source [24]. Furthermore, individuals who are wrong about the information or disseminate untrue details about a particular fact respond more positively to being corrected when they share a relational bond or community connection with the corrector [40]. Those engaged in misinformation correction prefer approaches that rely on relationship-building because those approaches allow them to demonstrate politeness and express emotions [41].

In this study, we explore source-based and relational approaches to addressing misinformation separately. Source-based and relational approaches are distinct activities, and research on the motivations or barriers to each is growing.

### Scientists’ engagement in science communication activities

Research shows that scientists who participate in communication activities will continue to participate in communicating about science with the public in the future [42,43]. In other words, communication begets communication. Research on scientists also suggests that participating in science communication is associated with increased self-efficacy, social norms, and positive attitudes about science communication activities [15,22].

Based on this evidence, we pose the following hypothesis:

H1: Higher levels of engagement in science communication activities are positively related to behavioral intentions to correct misinformation using (a) source-based strategies and (b) relational strategies.

### Relationship between engagement in science communication activities and willingness to correct misinformation

Scientists who have more experience in science communication activities may feel more concern about misinformation than scientists who have less experience. Through their engagement in science communication activities, scientists can broaden participation in tackling societal issues and improving public perception of science (e.g., public talks) [44,45]. Additionally, engaging in science communication helps scientists contribute to shaping the direction of political and policy decisions by educating citizens about the challenges affecting the world [45]. Concern towards misinformation may predict scientists’ engagement in science communication activities and intentions to correct misinformation. Therefore, the following hypothesis is proposed:

H2: Higher levels of engagement in science communication activities are positively related to negative attitudes toward misinformation.

Previous studies have examined the association between individuals’ attitudes and their behavioral intentions to combat the spread of misinformation [16,46]. For instance, Ding et al.’s study found that positive attitudes toward COVID-19 rumor recognition (i.e., positive attitudes toward verifying information that people are skeptical about) and intentions to identify COVID-19 rumors are positively correlated [16].

Other studies have found a relationship between attitudes and behaviors around spreading misinformation online [17,18]. According to Khan and Idris, those who hold positive attitudes toward information verification are less likely to share unverified information on social media [17]. Pundir et al. found that attitudes toward news verification are positively related to the intention to validate news before disseminating it [18].

Although limited, findings from the studies discussed in this section indicate that attitudes toward misinformation are associated with a positive behavioral intention to correct misinformation. Indeed, research has shown that higher levels of perceived severity of misinformation are associated with intentions to correct misinformation [47]. Thus, the following hypothesis is proposed:

H3: Attitudes toward misinformation mediate the effect of engagement in science communication activities on behavioral intentions to correct misinformation using (a) source-based strategies and (b) relational strategies.

### Barriers to addressing misinformation

While trust and relationship-building are foundational to disseminating accurate, verified information and correcting misinformation, research is limited in these areas, especially on scientists. Instead, most research suggests that scientists embrace a deficit model attitude where they take a superior position when speaking *to* non-scientists. For example, Dudo and Besley found that scientists’ main objectives for public engagement are to “inform the public about science” and to “defend science from misinformation” [22]. Scientists’ lowest priorities are to strengthen the public’s trust in science (i.e., build trust) and to connect to their audience through science stories (i.e., tailor messages) [22].

#### The role of deficit model perception on behavioral intention to correct misinformation

There are multiple positionalities by which scientists can communicate with non-scientists. These can be divided into “traditional” models that focus on transmitting science to non-expert audiences and “non-traditional” models that focus on discussions involving knowledge outside of science [48]. These models have been further sub-categorized by various authors, such as Brossard and Lewenstein’s description of deficit/science literacy, contextual, lay expertise, and public participation models [49]. Trench described the deficit/dissemination, dialogue, and conversation/participation models [50]. Akin and Scheufele outlined the deficit model, dialogue model, and communication in context model [51]. Regardless of specific terminology in these and other studies characterizing science communication models, deficit-based models assume non-scientist publics are ignorant or monolithic, while more participatory models of science communication tend to focus on eliciting multiple perspectives from individuals and groups with diverse identities and expertise.

The deficit model has been criticized because it unnecessarily characterizes those with concerns about science as ignorant or lacking adequate knowledge about science [52,53], yet it remains persistent in science communication efforts [52,54]. An analysis of 515 science engagement and outreach activities in Australia found that most followed a mix of deficit or dialogue approaches and lacked inclusive/participatory approaches [55]. This is problematic because the deficit model assumes that scientific knowledge is superior to other worldviews [56], thus reinforcing racism and exclusion in science and STEM [57].

There are several reasons for the persistence of the deficit model. Science communication arose amid a model of one-directional communication to the public [54], with success defined as the diffusion of information from a sender to a receiver [58]. Additionally, science itself has been conceptualized as the production of knowledge that occurs separately from a society that is then informed of findings [54]. Furthermore, scientists lack training in communication and often see themselves as rational decision-makers while the public is some sort of deficient “other” [59,60]. Finally, STEM scientists’ lack of respect for social science can contribute to their deficit view of the public [60]. Scientists who have a positive attitude toward social sciences are more likely to stray from the deficit model of scientific opinion formation [60].

In a deficit model of science communication, experts need to only inform the audience and provide data and scientific facts in an understandable way. In a more dialogic model of science communication, scientific experts have responsibilities of “sharing input that is well received by others, listening to and learning the input of others, and investing in a relationship with others” [56]. Thus, utilizing non-deficit science communication approaches requires different skills on the part of scientists, and the science community needs additional training in these skills [52].

There are multiple ways that different science communication models can intersect with misinformation. In terms of communication about health, the move to more participatory approaches can build more one-on-one trusting relationships in science and health information; conversely, it could also lead to the rise of misinformation as scientific information is disseminated on social media and other sources instead of being filtered through experts [61]. When it comes to correcting misinformation, the deficit model assumes that any public skepticism in science is due to a lack of knowledge and can be solved with more information [62], suggesting that literacy- and source-based approaches to correcting misinformation may be appropriate. Conversely, more dialogic models recognize the various contexts that may influence public distrust or skepticism of science [62], suggesting that contextual and relational approaches to correcting misinformation may be more effective. Thus, there are complex interactions between science communication models and the rise, dissemination, and correction of misinformation.

**Fig 1:**
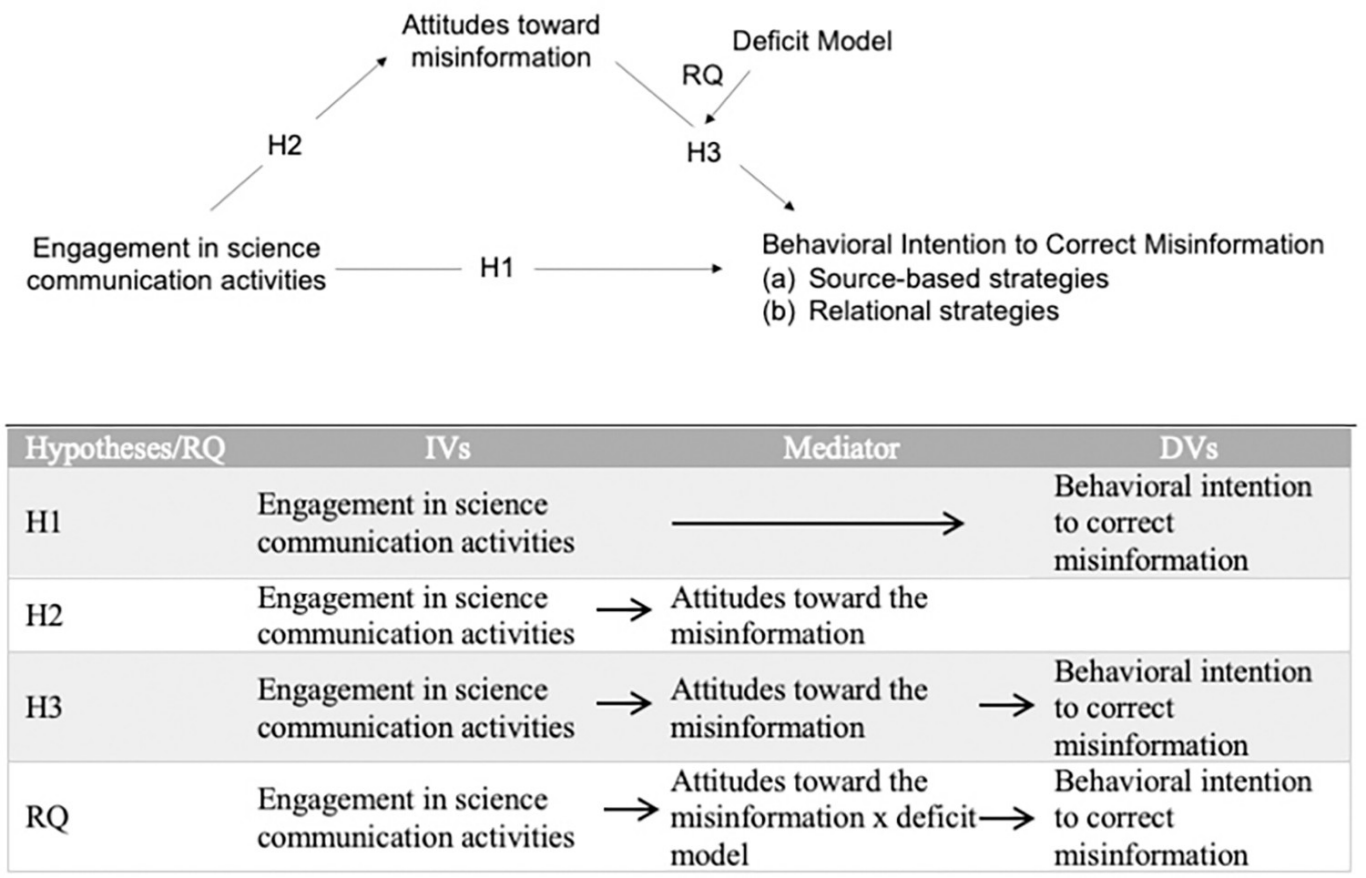
Conceptual model of the study with hypotheses.

In this study, with a focus on scientists themselves and their communication activities, we were especially interested in how scientists’ perceptions of the public and how strongly they see the public as a deficient “other” [60] influence their desire to correct misinformation. Fig 1 presents the conceptual model for the following research question.

RQ1: Do the direct and indirect effects of engagement in science communication activities on behavioral intentions to correct misinformation using (a) source-based strategies and (b) relational strategies differ based on the level of the deficit model perception?

## Method

### Ethics statement

This study and its consent procedure were approved by the Institutional Review Board at Colorado State University (IRB: #3219) where the authors work. Before beginning the online survey via Qualtrics, all participants provided written informed consent. The personal identity information (e.g., IP address) for all respondents was not collected. Participants consented to sharing their data in the aggregate and not as individual responses. To uphold the ethical guidelines of our Institutional Review Board and respect the privacy of our participants, we cannot share the data set in a public repository. Data requests may be sent to the Colorado State University Institutional Review Board (CSU_IRB@colostate.edu).

## Procedures and participants

This study utilized a geographically diverse sample of U.S. academic scientists from 25 land-grant universities. Within the six accreditation regions of the Council for Higher Education [63] in the United States (i.e., New England, Middle States, North Central, Southern, Western, and Northwest), four land-grant institutions were randomly chosen from each region.

The sample was also stratified by type of land-grant university. In the United States, there are three types of land-grant institutions [64]. (1) the original 57 public universities of 1862, which comprise more than 50% of the land-grant universities (57 out of 112); (2) the 19 historically Black colleges and universities that were added 1890; and (3) the 36 tribal colleges and universities that were added in 1994 [64]. For each accreditation region, two institutions were randomly chosen from the 1862 group, one institution was randomly chosen from the 1890 group, and one was randomly chosen from the 1994 group. If there were no institutions from the 1890 group in a region, an institution from 1994 was randomly chosen and vice versa. If both the 1890 and 1994 groups were not represented in an accreditation region, all four institutions were randomly chosen from 1862 group.

Within each land-grant university, six departments were chosen: five departments were randomly chosen from STEM (science, technology, engineering, and mathematics) fields based on the U.S. National Science Foundation (NSF) list [65], and one department was randomly chosen from the agriculture field. In addition, the authors’ land-grant university was added to the institution list.

Five research assistants visited the websites of selected departments to compile a list of faculty and researchers. Then, using manual search methods, the assistants gathered the email addresses of these individuals from each institution’s website and recorded them in a database. A total of 5,184 emails were obtained from 24 land-grant universities. Between May and June 2022, potential respondents received an initial invitation followed by two reminders. The survey was also distributed over a listserv that included all academic researchers and faculty at the authors’ institution.

Although 454 responses were collected, not all respondents answered all the questions. Respondents (*n* = 38) who only answered the first section of the questionnaire were eliminated from the sample, leaving a final sample size of 416. In addition, the data contained 13% missing data in key variables for analysis, and we assessed the structure of the missing values using the visualization method [66,67]. As the visualization inspection showed unstructured missing data patterns with no evident mechanism, missing data were handled using hot-deck imputation with the VIM package using R. Hot-deck imputation allows missing data (recipient) to be replaced by similar data that do not have any missing data (the donor) [66,68]. Therefore, this method allows all remaining data of 416 responses to be employed.

Of the 416 responses, 218 were males (52.4%) and 168 were females (40.3%), followed by those who said they prefer not to disclose (6%) and non-binary (1.2%). The scientists who responded were from life sciences (22.6%), social sciences (21.2%), math/computer sciences (14.9%), physical sciences (13.2%), humanities (12.3%), agriculture (8.7%), and material sciences and engineering (7.2%). When asked about the decade that they received their highest degree, respondents responded that they received it in the in the 2020s (15.9%), in the 2010s (29.6%), in the 2000s (22.4%), in the 1990s (18.3%), or prior to the 1980s (13.9%). The majority of scientists reported themselves to be White (76.2%) followed by those who indicated “prefer not to answer” (6.7%), mixed ethnicity (5.8%), Hispanic (4.8%), and Asian (4.1%). The remainder of the respondents indicated they were either Black, Native American, or other (2.4%).

### Measurements

#### Engagement in science communication

Engagement in science communication was measured by five statements that included: “During the past 5 years, about how often have you participated in the following activities?”: (1) Met with local, state, or federal policymakers, (2) Participated in public meetings, deliberative forums, or science festivals, (3) Given an interview with a journalist, (4) Written a news article, press release, blog post, or op-ed, and (5) Posted about a scientific topic on social media [42]. A 5-point Likert-type scale was used ranging from “not at all” to “very often”; the data were combined into one variable (*α* = .70).

#### Attitudes toward misinformation

To measure respondents’ attitudes toward misinformation, they were asked “Misinformation can impact different areas of society. How concerned are you about the harm of misinformation to the following?”: (1) environment, (2) human health, (3) animal health, (4) political climate, (5) government, (6) news media, and (7) society as a whole (adapted from [69]). These items used a 5-point Likert-type scale ranging from “not very concerned” to “very concerned” and were combined into one variable (*α* = .85)

#### Deficit model perception

We adopted Yuan et al.’s scale to assess how scientists view the general public [70]. As the original items were designed to measure scientists’ attitudes toward the public, we adopted one item that was used to measure the extent to which scientists held a deficit model perception of the public. This item was “the general public has little knowledge about science.” A 5-point Likert-type scale was used ranging from “strongly disagree” to “strongly agree” (*M* = 4.05, *SD* = 1.05).

#### Behavioral intention to correct misinformation using relational strategies

Individuals’ intention to correct misinformation using relational strategies was assessed by asking respondents whether they would correct misinformation using relational strategies on social media and in one-on-one conversations. The questions included: “How likely are you to do the following to correct misinformation in the future? (1) use my own words to respond to a post on social media, and (2) talk with someone one-on-one.” A 5-point Likert-type scale was also used ranging from “extremely unlikely” to “extremely likely,’” and these two items were averaged into a single item (*α* = .71).

#### Behavioral intention to correct misinformation using source-based strategies

Individuals’ intention to correct misinformation using source-based strategies was assessed by asking “How likely are you to do the following to correct misinformation in the future? (1) provide corrective information from a government organization, (2) provide corrective information from news media, and (3) provide corrective information from schools, faith organizations, or other community organizations.” A 5-point Likert-type scale was used ranging from “extremely unlikely” to “extremely likely,” and these three items were averaged into a single item (*α* = .82).

#### Control variables

Previous studies, such as [71], have controlled for demographic variables, such as gender, race/ethnicity, and discipline. Therefore, in our analyses, we controlled for participants’ gender, race/ethnicity, and discipline.

#### Statistical analyses

To investigate hypotheses 1 and 2, we used hierarchical ordinary least square regression, which enabled us to enter variables in distinct blocks so that we tested the incremental assessment of *R*^*2*^ in each block as well as the relative effects of variables while taking into account those entered simultaneously or in earlier steps [72]. To test hypothesis 3 and the research question, we used PROCESS Model 4 and Model 14 (respectively) with 5,000 bootstrap iterations to evaluate the power of the unstandardized indirect effects. The PROCESS Macro [73] has been widely used to evaluate indirect effects in the mediator model.

## Results

We hypothesized that higher levels of engagement in science communication activities are positively related to behavioral intentions to correct misinformation using (a) source-based strategies and (b) relational strategies. As shown in Table 1, H1(a) and H1(b) were supported. Engagement in science communication activities was positively correlated with behavioral intentions to correct misinformation using source-based strategies (*β* = 3.53, *p <* .001) and using relational strategies (*β* = 5.47, *p <* .001).

**Table 1:** Results of regression analysis.

Predictors	Attitude toward misinformation		Behavioral intentions to correct misinformation using source-based strategies		Behavioral intentions to correct misinformation using relational strategies	
	*β*	*t*	*β*	*t*	*β*	*t*
Gender				.		.
Woman	0.169	3.557***	0.044	0.903	0.103	2.099*
Non-binary	0.031	0.668	0.083	1.777	0.108	2.29*
Prefer not to disclose	-0.15	-2.055*	-0.021	-0.292	-0.066	-0.89
Discipline						
Agriculture	-0.02	-0.42	0.045	0.962	-0.047	-0.994
Social Science + Humanities	0.09	1.9	0.104	2.165	-0.02	-0.418
Ethnicity						
Hispanic	-0.102	-2.185*	-0.039	-0.84	0.029	0.603
Asian	-0.027	-0.598	0.106	2.303	0.01	0.221
Black, Native, Hawaiian, and other	0.062	1.345	0.084	1.818	0.072	1.552
Prefer not to disclose	-0.149	-2.046*	0.024	0.329	0.039	0.53
Mixed	-0.026	-0.571	0.045	0.981	-0.021	-0.454
Engagement in science communication activities	0.092	2.007*	0.164	3.534***	0.256	5.467***
Attitude toward misinformation			0.255	5.115***	0.167	3.311**
*F*(p)	7.60***		6.86***		6.00***	
*R* ^*2*^	.17		.17		.15	
*adj*. *R*^*2*^	.15		.15		.13	

Reference group, Gender/Male, Ethnicity/White, Discipline/STEM

*p < .05

** *p* < .01

*** *p* < .001.

H2 predicted that engagement in science communication activities is positively related to attitudes toward misinformation. The results, as shown in Table 1, indicated that engagement in science communication activities is positively related to attitude toward misinformation (*β* = .10, *p <* .05).

Hypothesis 3 predicted that attitudes toward misinformation mediate the effect of science communication activities on behavioral intentions to correct misinformation using (a) source-based strategies and (b) relational strategies. As shown in Table 2, our results supported H3 (a) and (b). As predicted, the indirect effect via attitudes toward the misinformation was significant on behavioral intentions to correct misinformation using source-based strategies (indirect effect = .0435, 95% CI .0073, .0819) and using relational strategies (indirect effect = .0297, 95% CI .0046, .0608).

**Table 2:** Bootstrap results of the direct and indirect effects of engagement in science communication activities on outcome variables.

Results of mediation analysis	*β*	LLCI	ULCI
*Direct and indirect effects of engagement in science communication activities on behavioral intentions to correct misinformation using source-based strategies*			
Science communication activities → behavioral intentions to correct misinformation using source-based strategies	**.2125**	**.1032**	**.3218**
Science communication activities → attitudes toward misinformation → behavioral intentions to correct misinformation using source-based strategies	**.0297**	**.0074**	**.0807**
*Direct and indirect effects of engagement in science communication activities on behavioral intentions to correct misinformation using relational strategies*			
Science communication activities → behavioral intentions to correct misinformation using relational strategies	**.2968**	**.1899**	**.4037**
Science communication activities → attitudes toward misinformation → behavioral intentions to correct misinformation using relational strategies	**.0291**	**.0044**	**.0602**

*Note*: Coefficients are unstandardized.

Abbreviations: LLCI, lower level of confidence interval; ULCI, upper level of confidence interval.

Controlled for demographic factors and discipline. Significant effects in bold text.

**Fig 2:**
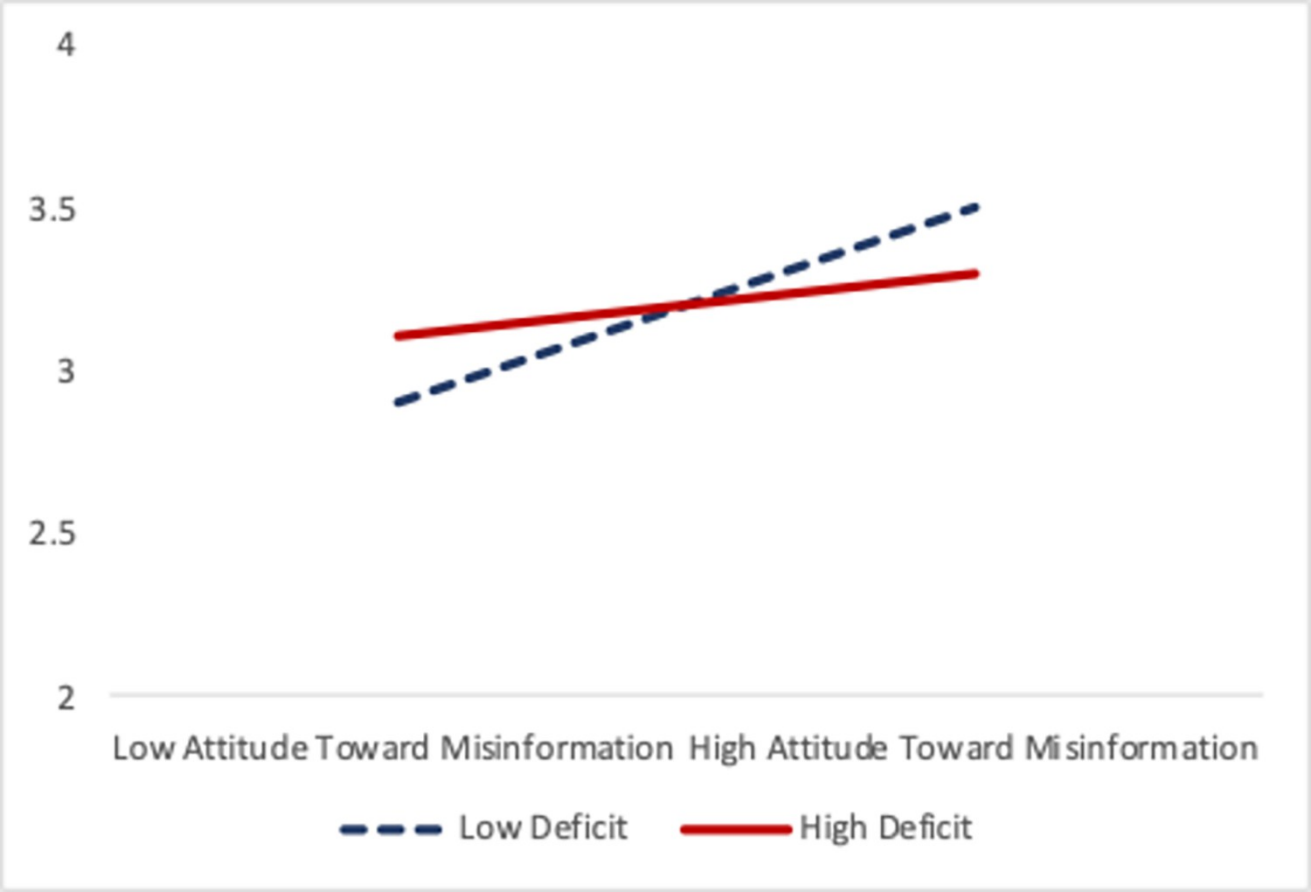
Interaction of scientists’ attitude toward misinformation and their deficit model perception on their intentions to correct misinformation using relational strategies.

A moderated mediation analysis was performed to test the research question about the deficit model perception. Model 14 from Hayes [73] showed a significant moderated mediation, suggesting a conditional indirect effect on behavioral intentions to correct misinformation using relational strategies (index = -.0114, *SE* = .0080, 95% CI -.0314, -.0003). For the condition of a low deficit model perception, attitude toward the misinformation mediated the effects of science communication activities on behavioral intentions to correct misinformation using relational strategies (indirect effect = .0374, *SE* = .0181, 95% CI.0064, .0777, significant as CI excludes zero). However, for the condition of a high deficit model perception, the mediation was diminished (indirect effect = .0146, *SE* = .0130, 95% CI, -.0063, .0441) and non-significant (overall model fit: adjusted *R*^2^ = .1309, *F* (4, 408) = 8.78, *p* < .001). See Tables 3 and 4, and Fig 2.

**Table 3:** Model coefficients for the conditional process model (process model 14).

	*Y* (Behavioral intentions to correct misinformation using relational strategies)		
Antecedent	*Coeff*.	*SE*	*p*
*X* (Science communication activities)	.2994	.0545	.0000
*M* (Attitude toward misinformation)	.6987	.2091	.0009
*W* (Deficit model perception)	.4657	.2260	.0400
*X* ✕ *W*	-.1112	.0529	.0364
Constant	-.5246	.9104	.5647

*R*^2^ = .1309 *F* (4,408) = 8.78, *p* < .001.

**Table 4:** Conditional indirect effect at specific levels of the deficit model perception.

Low deficit model perception	Indirect	Effect = .0374, *SE* = .0181, *95% CI* [.0064, .0777]
High deficit model perception	Indirect	Effect = .5011, *SE* = .1830, *95% CI* [-.0063, .0441]

On the other hand, the data showed that there is no significant moderated mediation of the deficit model perception on behavioral intentions to correct misinformation using source-based strategies (index = —.0066, *SE* = .0063, 95% CI -.0223, .0025).

## Discussion

The goal of this study was to understand how scientists’ communication experience and attitudes toward misinformation relate to their intentions to correct misinformation using two common strategies: source-based and relational approaches. Of note, our study identified an important potential barrier to the likelihood that scientists will engage in relational approaches: the deficit model perception. Despite increased sophistication in understanding how publics approach, interpret and respond to scientific issues, the deficit model–i.e., the idea that scientists need to simply transmit scientific knowledge to the public to increase understanding and support for science–persists. Therefore, our study raises important considerations for understanding what impedes scientists’ involvement in the pressing societal challenge of addressing misinformation.

### Limitations

Before we address the study findings further, it is important to note several limitations to our study. First, the study highlights the importance of the deficit model perception, which is a potential barrier to scientists’ likelihood to engage in relational approaches. However, utilizing a single-item measurement to capture the deficit model perception may not be sufficient. Multiple-item measures perform consistently better than their single-item equivalents [74]. Future work should consider exploring different dimensions of the deficit model perception. Furthermore, while the current study utilized self-reported survey data to explore factors that affect scientists’ use of source-based and relational strategies to address misinformation, the survey cannot confirm the causal relationships that were proposed in the conceptual model. Future studies should employ an experimental design to test these causal relationships. Last but not least, our sampling of scientists at land-grant universities limits the generalizability of the findings to scientists at other institutions. Scientists at land-grant institutions, however, do represent a group of scientists who may be particularly likely to participate in activities related to addressing misinformation, given that public engagement and outreach are central to the mission of such institutions. We also sampled widely across geographical regions and other characteristics, such as type of land-grant institution.

### Findings

Our study identifies how land-grant university scientists’ engagement in science communication activities contributes to their behavioral intentions to correct misinformation using source-based strategies and relational strategies. As an expected outcome, scientists’ higher levels of past engagement in science communication activities are positively associated with behavioral intentions to correct misinformation using source-based strategies and relational strategies. This finding is consistent with research showing that scientists’ public communication activities are related to other science communication activities [15,22]. Another expected outcome was that scientists who have more engagement in science communication activities perceive misinformation as more of a concern. Research in other contexts shows that increased involvement in communication activities, such as social media use, is positively associated with attitudes toward the same communication activity [75]. Scientists who have experience in science communication activities may have an increased awareness of prominent communication challenges in society, leading to their willingness to address those challenges.

Our results also show that scientists’ attitudes toward misinformation served as a significant mediator of the relationship between engagement in science communication activities and behavioral intentions to correct misinformation using source-based strategies and relational strategies. That is, when scientists were concerned about misinformation as a result of engagement in science communication activities, they were more likely to correct misinformation using source-based and relational strategies. This finding is supported by previous studies on the associations between attitudes and behavioral intentions. Past research has also shown that attitudes are an indicator of behavioral intentions in misinformation literature. For example, attitudes of social media users are positively associated with stronger behavioral intentions to recognize rumors in emergencies [16]. In addition, misinformation has been characterized as a prominent risk in society [69], and when people form higher risk perceptions on issues, they are more likely to act on them.

Our study revealed a moderated mediation relationship, such that engagement in science communication activities had an indirect effect on behavioral intentions to correct misinformation through attitude towards misinformation, and this relationship was moderated by the deficit model perception. Specifically, for scientists with a stronger belief in the deficit model, the indirect effect was weaker, indicating that their attitude towards misinformation played a smaller role in explaining the relationship between engagement in science communication activities and behavioral intentions to correct misinformation using relational strategies. That is, when scientists think the public knows little about science, they may be less likely to engage in relational conversations to address misinformation in their communities.

### Implications

It is possible that if scientists are not engaging in conversations with diverse members of the public, these members of the public may seek other spaces where their perspectives are heard, such as echo chambers where misinformation can spread [76]. Thus, coupled with public perceptions of uncertainties in science itself and a lack of consensus within the scientific community [77], scientists’ communication attitudes and behaviors can be a source of polarization. Scientists should instead grow as boundary spanners [78], recognizing their dual positionality as scientists and as members of their neighborhoods and other community groups. They have a responsibility to engage in respectful conversations about their community with fellow scientists and about science with community members. Greater attention should be paid to relational strategies for addressing misinformation. We suggest that providing science communication training within inclusive (rather than deficit model perception) worldviews and skills [79] would increase behavioral intentions to address misinformation relationally.

### Policy recommendations

Scientists’ engagement in addressing key science communication challenges, such as misinformation, has the potential to shape important societal outcomes. Misinformation negatively impacts human physical and mental health, social cohesion, and environmental systems [80,81]. Additionally, given the increased emphasis on communication shared in digital and social media communication environments, there are other communication problems that may exacerbate the impacts of misinformation. Problems such as cyberbullying can have a greater impact on those who are more socially vulnerable such as isolated elderly individuals [82] and adolescents [83]. Communication and human networks are part of the ecological system that enable positive health outcomes [84]. Encouraging greater engagement with communication among various publics in society, including scientists, will contribute to social change.

A number of factors go into developing capacities for scientists to engage in public communication activities, and many of those factors fall under organizational support for scientists. Training opportunities that promote inclusive, rather than deficit-model, science communication engagement should be built into curriculum for STEM students rather than as something outside of the degree-seeking experience [79]. Similarly, models of scholarly engagement that expand beyond traditional output (e.g., peer-reviewed publications) should be adopted at universities for scientific faculty. When scientists and STEM students are personally motivated to participate in science communication to address misinformation [85], institutions should provide directions and support for communication engagement that satisfies those motivations.

## Conclusions

This research study aimed to investigate the correlation between scientists’ communication activities, attitudes towards misinformation, the deficit model perception and their intentions to correct misinformation by employing source-based and relational strategies. The study emphasized that scientists’ engagement in science communication activities and their attitudes towards misinformation are significantly related to their behavioral intentions to correct misinformation using source-based and relational strategies. In addition, our results highlighted that the deficit model perception moderates the indirect effect of engagement in science communication activities on behavioral intentions to correct misinformation using relational strategies through attitude towards misinformation. In light of the urgent social challenge that misinformation poses, there is a need to focus more on employing effective strategies to address it. This calls for a collaborative effort among scientists, policymakers, and other stakeholders to establish science communication policies that promote training and adoption of relational strategies to address misinformation.
